# Signal complexity indicators of health status in clinical EEG

**DOI:** 10.1038/s41598-021-99717-8

**Published:** 2021-10-12

**Authors:** Kelly Shen, Alison McFadden, Anthony R. McIntosh

**Affiliations:** 1 Rotman Research Institute, Baycrest Centre, 3560 Bathurst Street, Toronto, ON M6A 2E1 Canada; 2 University of Toronto, Toronto, Canada

**Keywords:** Cognitive neuroscience, Diseases of the nervous system

## Abstract

Brain signal variability changes across the lifespan in both health and disease, likely reflecting changes in information processing capacity related to development, aging and neurological disorders. While signal complexity, and multiscale entropy (MSE) in particular, has been proposed as a biomarker for neurological disorders, most observations of altered signal complexity have come from studies comparing patients with few to no comorbidities against healthy controls. In this study, we examined whether MSE of brain signals was distinguishable across patient groups in a large and heterogeneous set of clinical-EEG data. Using a multivariate analysis, we found unique timescale-dependent differences in MSE across various neurological disorders. We also found MSE to differentiate individuals with non-brain comorbidities, suggesting that MSE is sensitive to brain signal changes brought about by metabolic and other non-brain disorders. Such changes were not detectable in the spectral power density of brain signals. Our findings suggest that brain signal complexity may offer complementary information to spectral power about an individual’s health status and is a promising avenue for clinical biomarker development.

## Introduction

A growing literature suggests that some degree of brain signal variability is vital to optimal brain function. Although seemingly paradoxical, noisy (or complex) brain signals are related to a greater capacity for information processing as compared to more predictable signals^1,2^. Sample entropy is one way to capture the variability of a brain signal^3^ and multiscale entropy (MSE), where complexity is examined across multiple timescales^4^, has been particularly useful in broadening our understanding of the role of noise in brain health and disease. MSE, like other measures of entropy, captures the variability in a signal but can additionally differentiate variability induced by increasing randomness, such that white noise has low MSE values^4^. MSE can therefore distinguish between the noise in brain signals induced by measurement and physiological variability. An increase in MSE has been observed in tasks requiring memory retrieval^5^ or the integration of stimulus features^6^ and seems to support accurate and stable behavior^6,7^. MSE has been shown to have timescale-dependent shifts during brain development^8–11^ and aging^12–14^ that supports cognitive function^15,16^, reflecting changes in the brain’s information processing capacity across the lifespan. MSE also reflects processing capacity changes related to various brain diseases including stroke^17^, dementia^18–20^, neurodevelopmental disorders^21–23^, and psychiatric disorders^24–26^.

In nearly all of these studies, brain signal complexity changes related to various brain diseases have been detected by comparing individuals with few to no comorbidities against matched healthy controls using data collected in highly controlled laboratory environments. While MSE has been proposed for use as a clinical biomarker for various neurological disorders^27–29^, whether differences in brain signal complexity can be detected across a heterogenous clinical population per se remains unknown. In this study, we leveraged the Temple University Corpus EEG database^30^ to test the utility of MSE as an indicator of health status in a large and heterogeneous clinical population. We found MSE of clinical-EEG signals differentiated various brain disorders. Interestingly, we also found MSE to differentiate between individuals with non-brain comorbidities and those without comorbidities.

## Methods

### Subjects

Clinical EEG data and corresponding physician reports were downloaded from the Temple University Hospital EEG Epilepsy Corpus (v0.0.1) containing 100 subjects deemed to have epilepsy and 100 subjects without epilepsy (https://www.isip.piconepress.com/projects/tuh_eeg/). ^30^. Subjects from the epilepsy group were included in our sample if the report indicated a previous diagnosis of epilepsy, if the EEG supported a diagnosis of epilepsy, or if the patient had experienced 2 or more unprovoked seizures occurring more than 24 h apart and the EEG did not contraindicate epilepsy. Subjects without epilepsy were included if they did not meet any of these criteria. Subjects from either group were excluded if a seizure occurred during the recording, if the subject’s level of consciousness was decreased, or if the subject was under the effect of a device likely to cause substantial EEG artifact such as a pacemaker or ventilator. Subjects were also excluded if their recordings were deemed unsuitable in the preprocessing stage due to the presence of artifacts.

Demographic and clinical characteristics were extracted from the physician reports. For the various brain-acting medications (anti-epileptic drugs, barbiturates, benzodiazepines, antipsychotics, and antidepressants), subjects were considered to be on them if their medication list included at least one medication of that category. The total number of other (i.e., not brain-acting) medications for each subject was computed by counting the number of total medications listed for the subject and subtracting the number of medications that fell into the brain-acting medication categories listed above. If the medication list stated “others” or a pluralized general category of medications (i.e. “antihypertensives”), two medications were added to the non-brain medication count. Most of the non-brain-acting medications reported (69.3%; 223/322) are those used to treat cardiovascular disease, diabetes or chronic respiratory illness.

Seizure classifications and terms were determined as outlined by the International League Against Epilepsy^31,32^. A subject was considered to have experienced generalized or focal seizures if their physician’s report contained either a diagnosis falling in one of those categories or a description of seizures matching the expected presentation for that seizure classification. Thirty-four subjects experienced seizures of unknown classification and were excluded from analysis.

Brain disorders were grouped into broad classes to capture the range of disorders in the heterogeneous sample while maintaining reasonable sample sizes within each category. Accepted phrases for stroke included indication of a past or present ischemic stroke, hemorrhagic stroke, “CVA”, or intracerebral bleed. Accepted diagnoses for degenerative brain diseases included Alzheimer’s disease, Parkinson’s disease, and dementia. Accepted diagnoses for psychiatric disorders included anxiety, depression, bipolar disease, and schizophrenia. Accepted diagnoses for neurodevelopmental disorders included Down’s syndrome, ADHD, intellectual disabilities, and cerebral palsy. Finally, the other brain disorders and injuries group included patients that had not yet received a brain-related diagnosis at the time of data collection (48.1%; 13/27), patients that had experienced acute brain trauma (e.g., head trauma, hypoxic/anoxic injury; 25.8%; 7/27) or brain surgery (e.g., craniotomy, resection; 25.8%; 7/27).

Age or sex information was not available for three subjects and they were excluded from analysis. This resulted in a total sample size of 163 subjects.

### EEG preprocessing & analysis

To allow for comparison with existing studies of MSE from EEG data collected in the laboratory, we treated the clinical-EEG recordings as if they were “resting-state” data by following methods that have been previously described^12,13^. Processing steps included epoching, bandpass filtering, rejection of trials with stimulation or excessive signal amplitude, and ICA-based artifact removal.

Each subject contributed one EEG recording. For subjects with multiple recordings, the recording corresponding to the physician report containing the most complete clinical picture was selected. For recordings that were split into multiple segments, the longest of the segments was chosen for preprocessing. All preprocessing was performed using the FieldTrip toolbox in MATLAB (www.fieldtriptoolbox.org)^33^. For each selected recording, 19 scalp electrodes of the International 10–20 system that were common to all subjects were selected. These were electrode positions Fp1, Fp2, F3, F4, C3, C4, P3, P4, O1, O2, F7, F8, T3, T4, T5, T6, Fz, Cz, and Pz. The resulting continuous recordings were segmented into 4-s trials, producing an average of 317 trials per subject, and bandpass filtered (Butterworth; 0.5 to 55 Hz). The majority of recordings were sampled at 250 Hz, but one subject that was sampled at 512 Hz was downsampled to 250 Hz before proceeding.

Two trial removal steps were then completed. The majority of subjects received photic stimulation. For these subjects, trials where photic stimulation began and ended were detected, and the trials within this range to 5 trials past the end of stimulation were removed. Trials at the beginning of a recording where the amplitude of the photic channel was not zero were also removed. Next, trials with excessive signal amplitude were detected for removal. For each subject, 30% of the trials that were determined by visual inspection to be reasonably free of artifacts were selected. Global field power was calculated and its mean ± 5 std was used to reject trials with time points outside of this threshold. The average number of remaining trials per subject following both of these removal steps was 178.

Independent component analysis was next used to remove ocular and muscle artifacts. Components with topographical distributions typical of these artifacts were selected and their traces further examined. Where possible, probable ocular artifact components were confirmed via alignment of the component trace with the electrooculogram traces from the original recording. Probable muscle artifact components were confirmed by the presence of a high frequency component trace. Finally, any recordings not referenced to a common average were re-referenced.

MSE^4,34^ was computed by first coarse-graining the EEG time series of each trial into 20 scales. To produce the time series coinciding with a given scale *t,* data points from the original time series within non-overlapping windows of length *t* were averaged. Thus scale 1 represents the original time series, with 1000 data points per channel per trial resulting from 4 s of recording sampled at 250 Hz. Next, sample entropy was calculated for each time series across all scales. This measured the predictability of the amplitude between two versus three consecutive data points (m = 2), with the condition that data points were considered to have indistinguishable amplitude from one another if the absolute difference in amplitude between them was ≤ 50% of the standard deviation of the time series (r = 0.5). The resulting values were averaged across trials to produce a single MSE curve per channel for each subject. As an entropy-based measure, MSE values are low for both completely deterministic as well as completely uncorrelated signals. We use the terms “finer” and “coarser” as relative descriptors for the range of timescales of MSE but there is no hard cutoff that distinguishes what is considered a finer or coarser timescale.

Spectral power (SPD) was calculated for each trial using the fast Fourier transform with a Hann window. To account for age-related global signal power changes, each recording was first normalized (mean = 0, *SD* = 1). Relative spectral power was then calculated for each trial, and results averaged across trials to acquire mean SPD per channel for each subject.

### Partial least squares analysis

MSE and SPD measures were each correlated with the available demographic and clinical data using a Partial Least Squares (PLS) analysis^35,36^. This multivariate statistical approach identifies a set of latent variables (LVs) that represent the maximal covariance between two datasets. First, the correlation between the MSE/SPD and clinical data was computed across subjects. Singular value decomposition was then performed on the correlation matrix to produce LVs, each containing three elements: (1) a set of weighted “saliences” that describe a spatiotemporal brain pattern of MSE/SPD measures; (2) a scalar singular value that expresses the strength of the covariance; and (3) a design contrast of correlation coefficients that express how the clinical data relate to the saliences. The mutually orthogonal LVs are extracted in order of magnitude, whereby the first LV explains the most covariance between MSE/SPD and clinical data, the second LV the second most, and so forth. We report the relative percentage of total cross-block covariance explained by each LV, where the sum of this percentage across all LVs is 100. The significance of each LV was assessed with permutation testing by randomly reordering subjects’ MSE/SPD pairing with clinical data to produce 1000 permuted sets for singular value decomposition, with the set of 1000 singular values forming the null distribution. The reliability of the MSE/SPD at each electrode in expressing the covariance pattern of each LV was assessed using bootstrap resampling. A set of 500 bootstrap samples was created by resampling subjects with replacement. The ratio between the saliences and the estimated standard error (bootstrap ratio) was taken as an index of reliability. With the assumption that the bootstrap distribution is normal, the bootstrap ratio is akin to a Z-score and corresponding saliences are considered to be reliable if the absolute value of their bootstrap ratio is >= 2. For the clinical data, confidence intervals were calculated from the upper and lower bounds of the 95th percentile of the bootstrap distribution of the correlation with the scores from the MSE/SPD data. The scores are the dot-product of the saliences with the data for each subject and are similar to a factor score from factor analysis.

For the demographic and clinical data entered into the PLS analysis, age and number of non-brain medications were treated as continuous variables, while all other variables were categorical. Sex was coded as 0 (M) and 1 (F). The remaining variables were coded as 0 (not on drug or does not have condition) or 1 (on drug or has condition).

**Figure 1:**
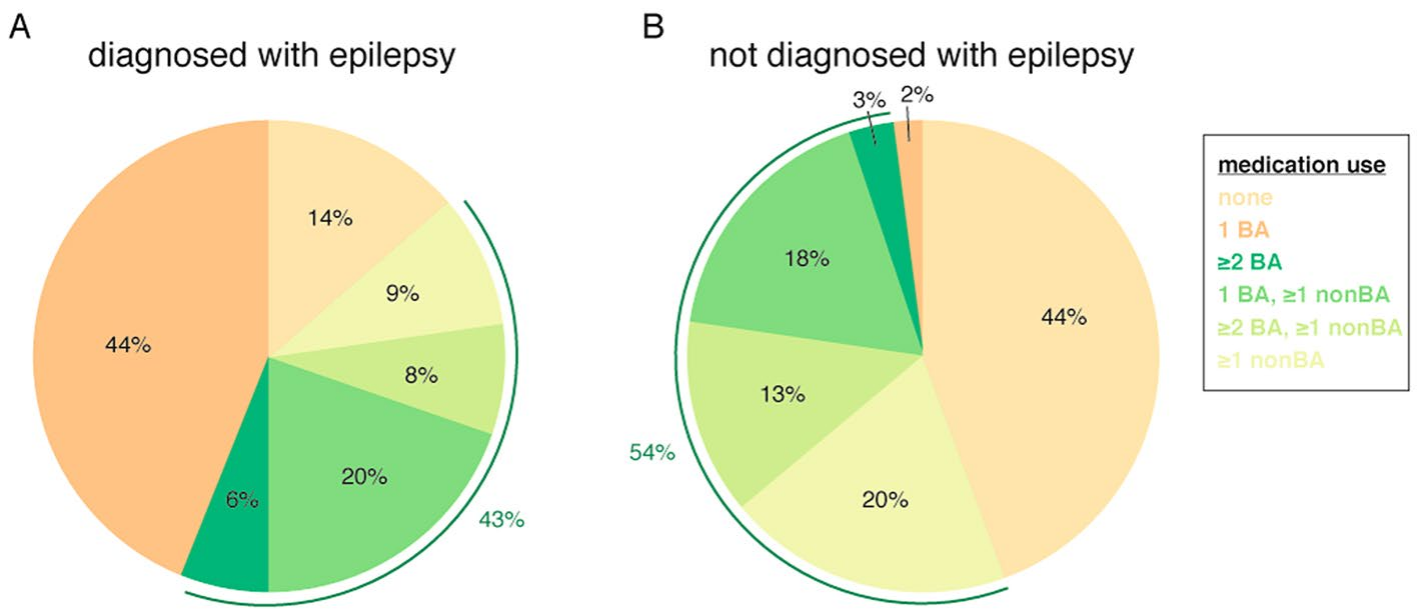
Brain- and non-brain-acting medication use in study sample. (**A**) Medication use in patients diagnosed with epilepsy (N = 66); (**B**) Medication use in patients without a diagnosis of epilepsy (N = 97). Indicators of comorbidities (use of multiple brain-acting medications, non-brain-acting medications, or both) shown in green. BA: brain-acting; nonBA: non-brain-acting. Figure panels created in MATLAB v.9.1 and merged using Adobe Illustrator CS6; colors were also edited in Illustrator for clarity.

Note that because the data from the Temple University Hospital EEG Corpus were collected for clinical purposes, the controls that are commonly a part of lab-based study designs (e.g., time of day of recording) cannot be considered or accounted for in our analysis. All analyses were performed and individual figures created using custom code in MathWorks MATLAB v.9.1 (https://www.mathworks.com/products/matlab.html). Multi-panel figures were then created and labelled using Adobe Illustrator CS6 (https://www.adobe.com/ca/products/illustrator.html). Editing of colors in Fig. 1 was also performed in Illustrator to enhance clarity.

## Results

Demographic and clinical characteristics of the study sample are summarized in Table 1. Briefly, this heterogeneous sample spanned a large age range (7–91 years) with slightly more female (~ 56%) than male patients. Comorbidities were present in a proportion of the patients, with ~ 21% of patients having more than one brain-related diagnosis, and ~ 15% were on more than one brain-acting medication. Nearly half (~ 48%) were on non-brain-acting medications. There were more slightly more female than male patients in all but one brain disorder group. Each group had a wide-ranging span of ages, with the smallest range being over three decades. Figure 1 illustrates medication use by patients with and without a diagnosis of epilepsy separately. A substantial proportion of patients in each group were either on multiple brain-acting drugs, on other non-brain-acting medications, or both (Fig. 1, green). These data together indicate that many patients were on multiple medications and/or had multiple diagnoses.

**Table 1:** Demographic and clinical characteristics of study sample.

*Variables*	*Subjects (n* = *163)*
Age, mean (SD, range)	52.12 (19.88, 7–91)
Sex, *n* female (%)	91 (55.83)
**Medication use**	
Brain-acting use, %	52.76
% of those with diagnosis of epilepsy	77.27
% of those without diagnosis of epilepsy	36.08
Anti-epileptic use, %	36.2
Barbiturate use, %	2.45
Benzodiazepine use, %	11.04
Antipsychotic use, %	9.82
Antidepressant use, %	11.04
**Past medical history (% female, mean age, age range)**	
Diagnosis of any brain disorder	71.78 (62.39, 51.19, 7–87)
Diagnosis of epilepsy, %	40.49 (66.67, 45.12, 7–82)
History of stroke, %	19.02 (61.29, 64.55, 32–87)
Diagnosed degenerative brain disease, %	4.91 (62.5, 66, 47–84)
Diagnosed psychiatric disorder, %	12.27 (60, 59.1, 35–83)
Diagnosed neurodevelopmental disorder, %	3.68 (66.67, 42.5, 19–75)
Other brain disorder or injury, %	16.56 (40.74, 45.52, 19–79)
**Indicators of comorbidity**	
Diagnosis of > 1 brain disorder, %	21.47
Use of > 1 brain-acting medication, %	15.34
Non-brain-acting medication use	
% of study sample	47.85
Mean number of medications (SD, range)	2.26 (2.75, 0–13)

Brain-acting medications include anti-epileptics, barbiturates, benzodiazepines, antipsychotics, and antidepressants.

**Figure 2:**
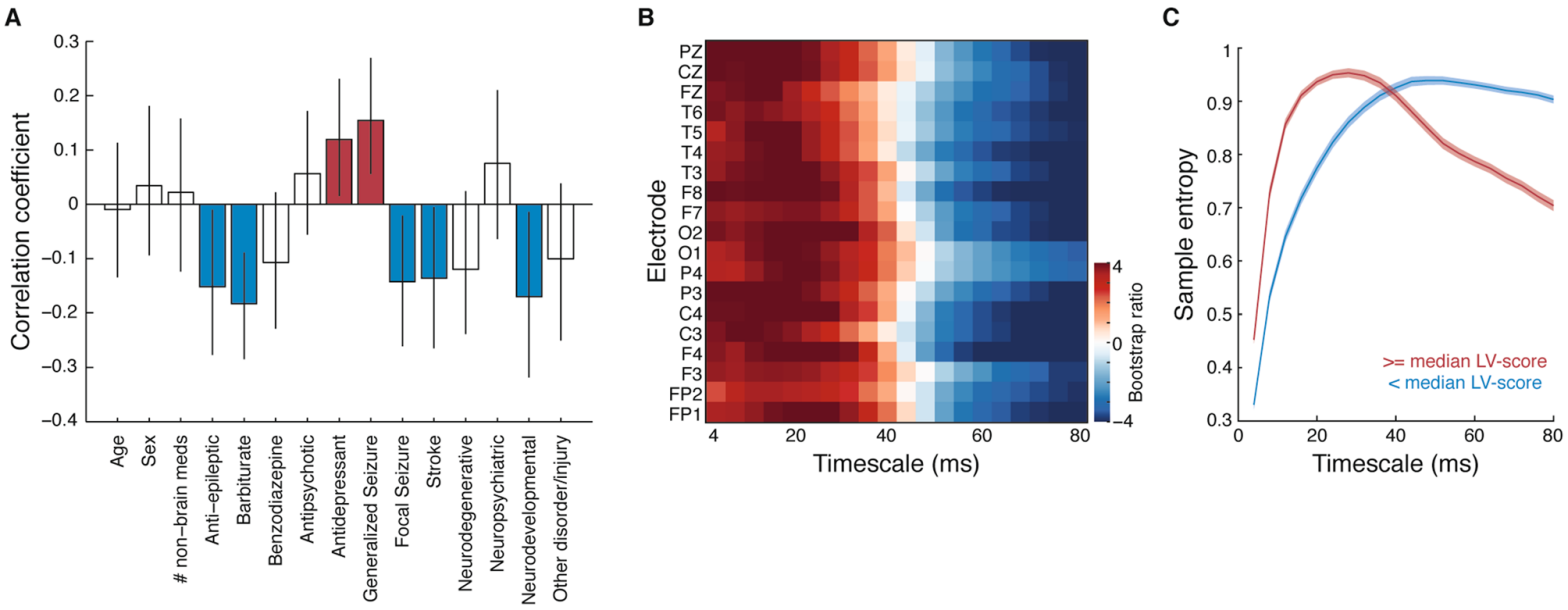
Brain signal complexity differentiates brain disorders. (**A**) Correlation coefficients and (**B**) bootstrap ratios of the first latent variable relating clinical data to MSE curves. (**C**) Average (± SEM) MSE curves, with subjects split into two groups according to their LV-scores. MSE curves were first averaged across electrodes within subjects, then averaged across subjects within each group. In (**A**), variables whose coefficients are significantly different from 0 are indicated in color for ease of interpretation. Figure panels created in MATLAB v.9.1 and merged using Adobe Illustrator CS6.

To determine whether different and heterogeneous clinical profiles can result in differences in brain signal complexity, MSE curves for each subject were correlated with their demographic and clinical data using a PLS analysis. The singular value decomposition of the correlation matrix resulted in two significant LVs. The first LV showed a differentiation between brain disorders, with a global shift towards greater signal complexity in finer time scales and lower signal complexity in coarser time scales across all electrodes for subjects who experienced generalized seizures or those taking antidepressants as compared to those with other brain conditions (i.e., focal seizures, stroke, neurodevelopmental disorders) or using other medications (i.e., anti-epileptics, barbiturates) (Fig. 2A,B). This shift in MSE was evident when a median-split was performed to classify subjects according to how much they expressed the patterns of the LV (i.e., a median split of the LV-scores, Fig. 2C). This LV was significant (p < 0.001) and accounted for 57.8% of the covariance across blocks.

**Figure 3:**
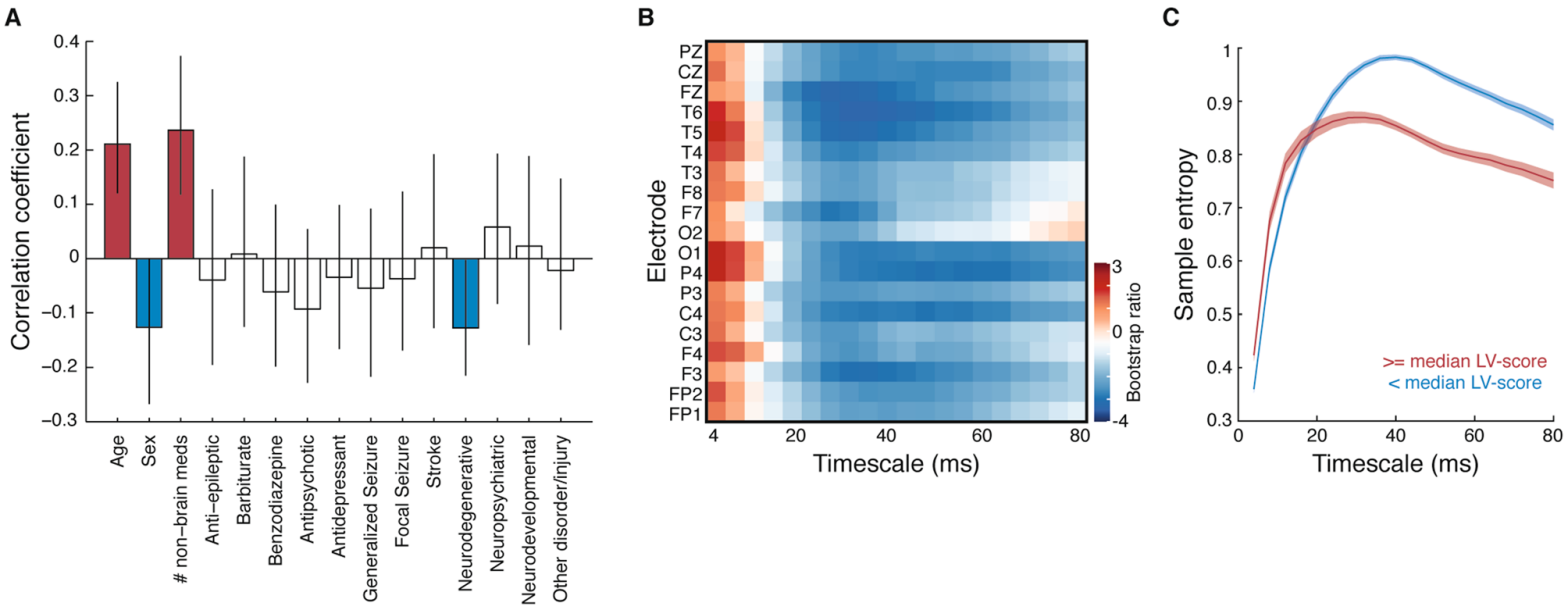
Brain signal complexity differs for older unhealthy males. Correlation coefficients (**A**) and bootstrap ratios (**B**) of the second latent variable relating clinical data to MSE curves. (**C**) Average (± SEM) MSE curves, with subjects split into two groups according to their LV-scores. MSE curves were first averaged across electrodes within subjects, then averaged across subjects within each group. In (**A**), variables whose coefficients are significantly different from 0 are indicated in color for ease of interpretation. Figure panels created in MATLAB v.9.1 and merged using Adobe Illustrator CS6.

The second LV differentiated older unhealthy (as indexed by the number of non-brain-related medications taken) males who did not have neurodegenerative disease from other subjects, and was associated with slightly higher entropy at the very finest scales and lower brain signal complexity across more coarse time scales (Fig. 3). This LV was significant (p < 0.01) and accounted for 29.0% of the covariance across blocks. The MSE profiles for each of the latent variables therefore reflected a unique timescale-dependent shift in brain signal complexity associated with different brain and non-brain disorders.

**Figure 4:**
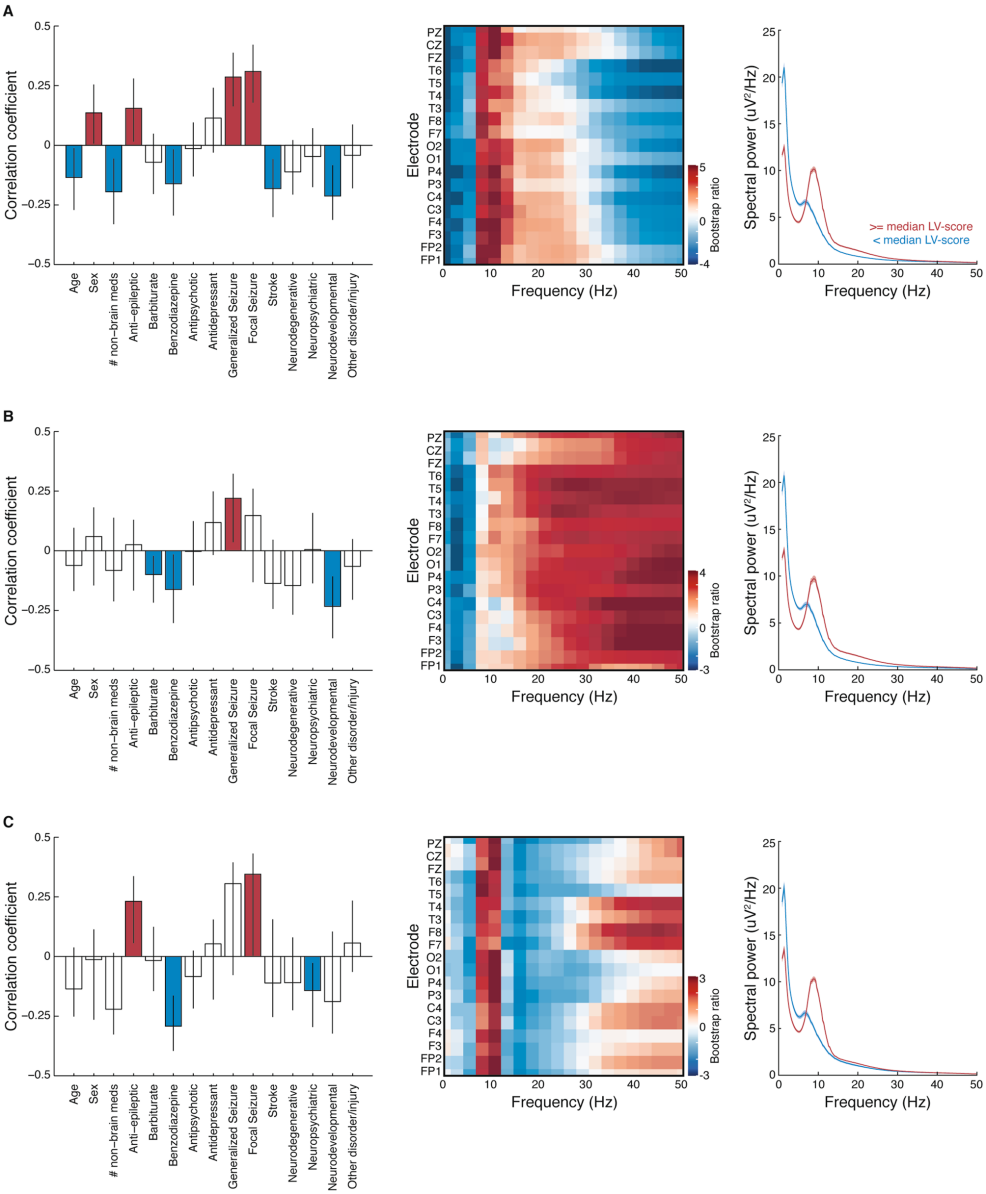
Spectral power density differentiates epilepsy from other brain disorders. (**A**) First latent variable (p < 0.001; 50.1% cross-block covariance) (**B**) second latent variable (p < 0.001; 29.9% cross-block covariance), and (**C**) third latent variable (p = 0.068; 10.1% cross-block covariance) of a PLS analysis relating clinical data to SPD. Left panels: correlation coefficients; middle panels: bootstrap ratios; right panels: average (± SEM) SPD, with subjects split into two groups according to their LV-scores. SPD functions were first averaged across electrodes within subjects, then averaged across subjects within each group. Figure panels created in MATLAB v.9.1 and merged using Adobe Illustrator CS6.

Changes in MSE can occur with changes in spectral power^8,37^ so a similar analysis of SPD was performed. The singular value decomposition of the correlation matrix between SPD and the demographic and clinical data resulted in two significant and one marginally-significant LV. All three LVs differentiated between subjects with epilepsy from those without epilepsy. In the case of the first LV, the differentiation was between younger female patients with epilepsy and older unhealthy male patients with other neurological disorders (Fig. 4A). The other two LVs captured patients who experienced generalized (Fig. 4B) and focal seizures (Fig. 4C) in contrast to neurodevelopmental and neuropsychiatric diagnoses, respectively. Despite the first LV capturing a subset of unhealthy male patients, similar to that from the MSE analysis (Fig. 3), the SPD profiles themselves were highly similar across LVs, with a decrease in power the delta and theta ranges and an increase in power in the alpha, beta and gamma range for patients with epilepsy. The SPD profiles could not therefore differentiate the patients with non-neurological comorbidities (e.g., metabolic disease) from those without those comorbidities.

## Discussion

In this study, we examined whether brain signal complexity varied across individuals of a large and heterogeneous clinical population using a data driven approach. We found timescale-dependent differences in brain signal complexity for individuals who experience generalized seizures from individuals who have other brain disorders (e.g., focal seizures, stroke, neurodevelopmental disorders). We also found a timescale-dependent shift in brain signal complexity for older males on various medications not related to neurological or neurodegenerative disease that was not uniquely evident in the spectral power of the clinical-EEG recordings. Our findings suggest that brain signal complexity, as indexed by MSE, can provide additional insights into brain health status and function not captured by spectral power.

In line with the notion that the brain is a dynamical system in which “noise” allows for flexible functioning and a variety of metastable states^38,39^, MSE can be considered as an index of functional repertoire^5^. Changes to brain function and dynamics can occur with neurological disease and, indeed, differences in MSE from matched controls have been reported for both epilepsy^40^ and neurodegenerative disease^28^. Here we build on these previous reports by showing how the changes in MSE in these neurological conditions can be differentiated from each other. A complementary data-driven analysis of SPD showed changes in power across frequency bands that differentiated epilepsy from all other diagnoses, consistent with numerous accounts of SPD changes in epilepsy when compared to healthy controls^41–45^. That the shifts in SPD between patients with epilepsy and other neurological patients are opposite to previous comparisons between patients with epilepsy and healthy controls^43,46^ suggest that there are differential changes to SPD in other neurological disorders as well. This is consistent with existing comparisons of these disorders with healthy controls^47–51^. Interestingly, MSE profiles differentiated patients with generalized seizures from those with focal seizures but SPD profiles did not. The differentiation of individuals with non-neurological comorbidities was also unique to MSE. While the dominant patterns in SPD detected by our statistical model did not differentiate patients along the same lines as MSE, it does not preclude the possibility that certain subgroups could be differentiated if a direct comparison of particular subgroups was performed.

The MSE results replicate previous observations that the scale-dependent changes are indicative of neurodegenerative disorders (Fig. 3). Higher MSE at coarse-scales was shown to predict cognitive decline in Parkinson’s patients who would later develop dementia^18^. The relative balance within subjects between finer and coarser scales also relates to cognitive status in aging^15^. These results, considered in the context of the present data, suggest that the relative shifts of complexity across temporal scales may be a sensitive index to assist in clinical evaluation, particularly as a predictor of future cognitive decline^52^. The demographic and clinical breakdown of the study sample, with its heterogeneity in medication use, comorbidities, age and sex across brain disorder groups suggests that any single variable in our model (e.g., age, sex, diagnosis) could not alone account for our findings. Instead, our data-driven approach suggests that, when considering all patients and all clinical profiles, the average MSE profiles for certain neurological conditions are similar (e.g., patients on antidepressants and those experiencing generalized seizures). It is possible that a different MSE feature may differentiate these groups of patients and further work is needed to determine the MSE features that are most valuable for the development of clinical tools.

Metabolic diseases such as diabetes mellitus are known to affect brain structure and cognitive function^53,54^. More recently, changes to resting-state functional networks have been observed in individuals with diabetes mellitus compared to controls^55^. Autonomic dysfunction, such as hypertension and heart failure, is also a well-documented risk factor for cognitive impairment^56–58^ and has been associated with changes to brain structure^59–61^ and function^62–64^. As such, both diabetes mellitus and hypertension have been linked to neurological disorders such as stroke^65^ and dementia^66^. One previous report has shown how hypoglycemic conditions in individuals with Type 1 diabetes mellitus results in changes to brain signal MSE^67^. We extend these previous findings by showing that the effects of various non-neurological diseases on the brain can be detected by MSE. Future work is needed to examine the mechanistic links between metabolic function and “noise” or information processing capacity of the brain.

We have demonstrated the dimensions along which MSE can differentiate clinical groups. An important next step will be to determine how well MSE performs within a predictive modelling framework. For example, machine learning approaches have great potential in clinical applications^68^. In combination with larger clinical datasets, including the full Temple EEG Corpus^30^, future work is needed to evaluate the utility of MSE in classifying and predicting patient populations. Together with evidence that MSE changes in response to medical therapies^69–72^, MSE offers a promising avenue for the development of clinical biomarkers.
